# Postpandemic Stress Disorder among Health Care Personnel: A Cross-Sectional Study (Silesia, Poland)

**DOI:** 10.1155/2022/1816537

**Published:** 2022-11-14

**Authors:** Mateusz Grajek, Patryk Szlacheta, Karolina Sobczyk, Karolina Krupa-Kotara, Beata Łabuz-Roszak, Ilona Korzonek-Szlacheta

**Affiliations:** ^1^Department of Public Health, Faculty of Health Sciences in Bytom, Medical University of Silesia in Katowice, 41902 Bytom, Poland; ^2^Department of Toxicology and Health Protection, Faculty of Health Sciences in Bytom, Medical University of Silesia in Katowice, 41902 Bytom, Poland; ^3^Department of Prevention of Metabolic Diseases, Faculty of Health Sciences in Bytom, Medical University of Silesia in Katowice, 41902 Bytom, Poland; ^4^Department of Health Economics and Health Management, Faculty of Health Sciences in Bytom, Medical University of Silesia in Katowice, 41902 Bytom, Poland; ^5^Department of Neurology, Institute of Medical Sciences, University of Opole, Opole, Poland; ^6^Department of Epidemiology, Faculty of Health Sciences in Bytom, Medical University of Silesia in Katowice, 41902 Bytom, Poland

## Abstract

**Background:**

Postpandemic stress disorder (PPSD) is an unofficial term that refers to posttraumatic stress disorder (PTSD), a mental disorder resulting from increased stress, anxiety, and trauma associated with unpleasant life experiences. Many scientific studies indicate that symptoms of increased stress, job burnout, anxiety, and depressive disorders are associated with medical personnel performing their professional duties around COVID-19 patients.

**Objective:**

The purpose of this study was to assess the prevalence of symptoms that may indicate the presence of PPSD symptoms—depression, anxiety, and stress—in medical personnel. *Material and Methods*. The survey included 300 people, representatives of medical personnel. The group was divided into two sections. The first section numbered 150 and consisted of personnel in direct contact with COVID-19 patients (FR); the second group also consisted of 150 medical professionals, who but no longer directly involved in helping with COVID-19 cases (SR). The survey was conducted by indirect survey method using CAWI (computer-assisted web interview). The survey used a questionnaire technique. A proprietary tool enriched with standardized psychometric scales: BDI, GAD-7, FCV-19S, and PSS-10 was used. Kruskal-Wallis and Mann–Whitney *U* statistical tests were used in the statistical processing of the data. The probability level was 0.05.

**Results:**

Statistical inference made it clear that mental health problems that may indicate trauma are mainly present in the FR group. These symptoms decreased slightly in comparison between periods 2020 and 2021 (*p* < 0.05).

**Conclusions:**

The COVID-19 pandemic significantly increased the prevalence of depression, anxiety, and stress among first responders. To ensure the psychological well-being of first responders, early assessment and care of mild depression, anxiety, and stress should be promoted to prevent the development of moderate and severe forms.

## 1. Introduction

The COVID-19 pandemic is, in many ways, the greatest worldwide epidemic in over a century, due to its widespread nature, severity, and complicated and long-term consequences. Apart from the virus's devastating direct health effects, other aspects of the pandemic—such as the fear of transmission, the consequences of interventions to reduce transmission, massive economic strain, societal disruption, and the loss of family and friends—undoubtedly have a complex impact on mental health [1, 2]. Millions of people have been impacted by COVID-19 worldwide, making it a legitimate threat to global health [3]. The first report of a patient with SARS-CoV-2 infection was made in Wuhan in December of 2019 [4]. Since then, the virus has spread throughout the world, resulting in a massive, unheard-of healthcare disaster. On January 30, 2020 and March 11, 2020, respectively, the World Health Organization (WHO) labeled the condition a Public Health Emergency of International Concern (PHEIC) and a pandemic [5]. Global COVID-19 mortality has surpassed 6 million as of today (September 2022), with more than 596 million confirmed cases [6].

Frontline healthcare personnel who treat and care for patients who have SARS-CoV-2 infection are at risk of experiencing psychological distress and other mental health symptoms due to this severe circumstance. The mental toll on this personnel may have been exacerbated by an increase in the number of confirmed or suspected cases, an excessive workload, extensive media coverage, a shortage of particular medications, and emotions of being undersupported, fragility, or loss of control [7, 8]. One of the major paradoxes of the COVID-19 pandemic was experienced by them; although the general public was required to stay at home and avoid all social interaction, healthcare professionals were required to carry out their duties in close contact with the virus and be continuously exposed to it [7, 9].

According to earlier research, clinicians experienced considerable psychological discomfort during the COVID-19 pandemic, including anxiety, sadness, insomnia, burnout, and psychological anguish [8–10]. In all relevant categories, frontline employment was an independent predictor of poorer mental health outcomes [11]. The COVID-19 effect has been reported to enhance frontline healthcare workers' risk of mental illness. According to some research, rates of likely posttraumatic stress disorder (PTSD) are as high as 39% and probable moderate to severe depression is as high as 43%, with up to 13% of employees reporting suicidal or self-harm thoughts [12]. According to a recent meta-analysis, the prevalence of PTSD symptoms is 20.2% and that of depression is 31.1% [13]. Due to these high rates, frontline staff who provide treatment to COVID-19 patients have mental health as a top priority on a global scale [14]. Studies link the COVID-19 pandemic to the high prevalence of likely mental illness [9, 12, 15].

The review study by Cabarkapa et al. [16] revealed that among frontline healthcare workers (HCWs) during the COVID-19 pandemic, fear of the unknown or contracting the disease was the most frequent source of stress. Increased workloads, wearing protective gear, rapidly evolving regulations, the strain of adhering to stringent protective measures, a lack of adequate personal protective equipment (PPE), and concern over infecting loved ones were additional sources of stress. According to this analysis, female nurses have the highest risk of developing mental health issues among HCWs since they frequently interacted with COVID-19 patients who were both suspected and confirmed. Not only the COVID-19 pandemic but also the 2003 SARS pandemic had a long-term impact on the mental health of HCWs, according to an earlier study [17].

The HCWs, particularly nurses, were repeatedly identified as the high-risk category for developing PTSD during the COVID-19 outbreak, according to recent studies [18, 19]. Not only does early detection of probable PTSD cases shield HCWs from psychological harm but it also makes early diagnosis and treatment simple [20, 21]. It is crucial to comprehend and understand frontline healthcare workers' stress and how it affects their mental health, particularly when PTSD symptoms are present during a pandemic.

In a global survey undertaken by Denning et al. in 2021 [22], it was discovered that over 60% of HCWs, particularly nurses and doctors, were suffering from considerable burnout. Their levels of burnout were correlated with how safe they felt at work. Overly demanding jobs with few resources can lead to burnout, which manifests as physical and mental tiredness [23]. When faced with the COVID-19 pandemic's increased workload and scarce resources, such as insufficient PPE [16], HCWs frequently develop occupational burnout. This may affect how they perceive the safety of their working environments.

Nearly 50% of emergency department HCWs experienced moderate to severe levels of personal burnout, according to Chor et al. [24], and emergency nurses had higher burnout levels than doctors. Emergency nurses' susceptibility to high levels of burnout was correlated with stress from being on the front lines in close contact with suspected or confirmed COVID-19 patients, physical distress from care burden with the prolonged wearing of PPE, social isolation, and weak social support. Uncertainty exists regarding the variations in burnout following the COVID-19 outbreak and its relationships with stress levels and PTSD symptoms.

During the postepidemic period, emergency nurses were at significant risk of developing PTSD [25]. A month after the MERS outbreak [26] and two months after the SARS outbreak [27], earlier research utilizing retrospective and cross-sectional methods showed that HCWs were most at risk for developing PTSD symptoms. According to a recent review study, HCWs working in emergency units were most likely to experience PTSD during COVID-19, with rates ranging from 2.1% to 73.4% as measured by the posttraumatic stress scale (PTSS) [28]. Recent studies using a prospective design found a trend of decreasing PTSD symptoms among HCWs during follow-ups following the COVID-19 outbreak [19, 29–31]. However, in the fourth month of the follow-up, 30% of HCWs showed clinically significant PTSD symptoms, according to Dufour et al. [29]. ICU nurses had more PTSD symptoms than non-ICU nurses, according to Steenkiste et al. [31]. The results suggest that HCWs who were exposed to the direct care of patients with COVID-19 were more likely to experience PTSD symptoms. Emergency workers are frontline workers in the screening and care of patients with COVID-19.

In addition to PTSD, the review study revealed that a sizable proportion of emergency nurses had moderate to severe burnout following a traumatic occurrence [25]. Recent research has indicated that a sizable portion of frontline health care workers has at least mild symptoms of burnout during the COVID-19 epidemic [24, 32]. Additionally, during the first three months of the COVID-19 outbreak, emergency nurses showed higher degrees of personal burnout than doctors [24]. Their levels of burnout may be influenced by physical discomfort from wearing PPE, occupational stress, or both [32]. Burnout among HCWs is also linked to stress from worry about infecting loved ones and being apart from family because of travel limitations [22]. In the context of COVID-19, nothing is known regarding the association between emergency nurses' degrees of burnout and PTSD.

Given the above, the purpose of this study was to assess the prevalence of symptoms that may indicate the presence of postpandemic stress disorder (PPSD) symptoms—depression, anxiety, and stress—in medical personnel. Detailed analyses focused on answering the questions:

1. The risk of covid-work-related depression is higher in those who have direct contact with patients with COVID-19
2. The risk of covid-work-related anxiety is higher in those who have direct contact with patients with COVID-19
3. The risk of covid-work-related stress is higher in those who have direct contact with patients with COVID-19

## 2. Materials and Methods

### 2.1. Materials

The survey included 300 people, representatives of medical personnel. The group was divided into two sections. The first section numbered 150 and consisted of personnel in direct contact with COVID-19 patients and included 20 doctors, 62 nurses, and 68 paramedics. For statistical order, this group was called “first responders” (FR) to signify the fact that these are the people directly involved in helping a person with COVID-19. The second group also consisted of 150 medical professionals, who but no longer directly involved in helping with COVID-19 cases: 18 doctors, 58 nurses, and 74 paramedics—the group was called “second responders” (SR).

The eligibility criteria for study participants assumed that the subjects were medical employees actively working on a full-time basis in one of the hospitals in the Silesian province (Poland). In addition, a preliminary psychological and psychiatric interview was conducted to exclude from the study individuals who have a history of or currently exhibit symptoms of mental disorders, such as anxiety disorders or mood disorders. Ultimately, the aforementioned number of people were qualified for the study, rejecting 34 people along the way who did not meet the inclusion criteria.

### 2.2. Methods

The survey was conducted by indirect survey method using CAWI (computer-assisted web interview). The survey used a questionnaire technique. A proprietary tool enriched with standardized psychometric scales, the Beck Depression Inventory (BDI), Generalized Anxiety Disorder Assessment (GAD-7), Fear of COVID-19 Scale (FCV-19S), and Perceived Stress Scale (PSS-10), was used. In the questionnaire questions, respondents answered questions about age, gender, nature of employment, length of service, and whether or not they worked on a COVID-19 patient. The questionnaire was conducted twice, the first time in the last quarter of 2020 and the second time in the last quarter of 2021. Both measurements were made on the same group of people. To avoid confusion between measurements, the questionnaires were properly coded.

In addition, to avoid forgery in the online form, the survey was sent directly to the respondent's email address, antifake/bot responders in the form of CAPTCHA keys were used, and the number and time of logins were checked. The survey was conducted under complete anonymity and respect for human dignity. Due to its survey nature, it did not require the approval of the Bioethics Committee (the Law on the Profession of Physician and Dentist).

### 2.3. Measurements

BDI included 21 questions about the basic symptoms of depression [33]. The questions were scored on a scale of 0 to 3, with 3 representing the highest intensity of a given symptom. The final score of the test consisted of adding up the points for each question and reading the degree of depression: 0-11, no depression; 12-19, mild depression; 20-25, moderate depression; and 26 and above, severe depression. Cronbach's *α* coefficient for the normalization sample was 0.84.

The study used the GAD-7 screening questionnaire used to determine feelings associated with a generalized anxiety disorder. It is a 7-item scale, based on a 4-point Likert scale, used to assess the level of anxiety, as well as to assess the risk of generalized anxiety syndrome. The questions in the questionnaire allow the respondents to assess their feelings of anxiety, tension, and nervousness, their ability to control these feelings, the ease with which they arise, and problems with relaxation. In each question, you can get from 0 to 3 points depending on the frequency of occurrence of a given phenomenon (0, not at all; 1, a few days; 2, more than half of the days; and 3, almost every day) within the last 14 days. A score of 5, 10, and 15 points indicate mild, moderate, and severe anxiety, respectively. A score of at least 10 points indicates a high probability of generalized anxiety syndrome [34]. For the normalization sample in our study, Cronbach's *α* was 0.82, which indicates the very good reliability of the test.

Moreover, the FCV-19S scale was used according to Ahorsu et al. [35] (Polish translation—Pisula and Nowakowska, [36]). Subscales are scored according to Likert scale assumptions, where 1 means disagreement with the statement and 5 means full agreement with the statement. The scale consists of 7 items, the maximum possible score is 35 points. To obtain the percentage value necessary for data analysis, the raw score was multiplied by the value of the calculated coefficient (≈2.857; the value of the coefficient was calculated based on the mathematical operation −100%/maximum possible score (35)). For the study, the verbal interpretation of the obtained results was adopted: 76-100% high level of anxiety; 56-75% moderate level of anxiety; 26-55% low level of COVID-19 anxiety; and <25% no COVID-19 anxiety. A Cronbach's *α* score of 0.88 was obtained for the normalization sample in the author's study, indicating very good test reliability.

The PSS-10 is used to assess the intensity of stress related to one's living situation over the past month. The scale is designed mainly for research purposes and can be used in practice, screening, prevention, and assessing the effectiveness of therapeutic interventions. Scores from 0 to 13 are considered low, while scores of 20 and above are considered high. Cronbach's *α* coefficient for the normalization sample was 0.84.

### 2.4. Statistical Analyses

Kruskal-Wallis (ANOVA) statistical tests were used in the statistical processing of the data between the study groups at periods 2020 and 2021. The probability level was 0.05. The study was preceded by a pilot study, which was conducted on a group of 30 patients, where respondents could assess whether they understood the questions contained in the questionnaire. It was estimated that 96.7% of respondents understood the question (average Cohen's Kappa 0.92).

## 3. Results

The survey was conducted by 300 people, which included 38 doctors, 120 nurses, and 142 paramedics. The study group consisted of 182 women (66.6%) and 118 men (44.4%). The average age of the respondents was 38 years (13 years). The seniority of the respondents ranged from 2-15 years (an average of 6.5 years).

In the following description of the results, the study groups were divided according to the previously described criteria into personnel in contact with the COVID-19 patient—first responders (FR)—and those without such contact—second responders (SR).

The assessment of depressive symptoms using the BDI between the study groups showed that in the first period (last quarter of 2020) there were 6% cases of moderate depression in the FR group, and in the second period (last quarter of 2021) there were 2% cases of moderate depression in the same group. Correspondingly, there were 2% cases of moderate depression in the SR group each during the same periods. For mild depression, it was estimated that this one was more common in the FR group in both periods—58% (2020) and 66% (2021)—compared to the same periods in the SR group—44% and 51%. There were no cases of severe depression in the study groups. Severity of symptoms of mild depression in the group of medics doing COVID-19 patient contact work was statistically significant (*H* = 11.863; *r* = 0.628; *p* ≤ 001). Similarly, it was statistically found that the presence of depressive symptoms decreased slightly between the 2020 and 2021 periods. This is observed, among other things, by an increased percentage of people showing no signs of depression on the BDI (*H* = 10.346; *r* = 0.583; *p* ≤ 001). The absence of depressive symptoms was found in about 40% of respondents (averaged value). Detailed results are summarized in Table 1.

Evaluation of generalized anxiety symptoms using the GAD-7 between the study groups showed that in the first time interval (last quarter of 2020) there were 12% cases of mild and 14% of moderate anxiety in the FR group, and in the second time interval (last quarter of 2021) there were 12% cases of mild and moderate anxiety in the same group. Correspondingly, during the same periods in the SR group, there were 30% cases of mild and 10% of moderate anxiety (2020) and 34% of mild and 6% of moderate anxiety (2021). Severe anxiety symptoms were observed in 16% (2020) and 10% (2021) of the FR group and 4% (2020) and 6% (2021) of the SR group. The association of severity of anxiety symptoms with the group of medics performing COVID-19 patient contact work was statistically significant (*H* = 12.864; *r* = 0.666; *p* ≤ 001). Similarly, the presence of depressive symptoms was statistically found to decrease slightly between 2020 and 2021 (*H* = 11.717; *r* = 0.621; *p* ≤ 001). Generalized anxiety symptoms were diagnosed in about 40% of the FR group subjects and only 10% of the SR group subjects—a relationship that also proved significant (*H* = 10.383; *r* = 0.621; *p* ≤ 001). The absence of depressive symptoms was found in about 30% of the subjects (averaged value). Detailed results are summarized in Table 2.

Using the FCV-19S scale, the level of anxiety associated with COVID-19 among subjects was defined as moderate among 57% of the FR group. In the SR group, anxiety was assessed at a mild level (30%) —*H* = 12.391; *r* = 0.6329; *p* ≤ 001Table 3.

Based on the PSS-10 scale scores, it was found that the level of perceived stress also changed over time. In the FR group in 2020, 32% of the subjects showed a light level of stress while 28% in 2021. In the SR group, 44% of the subjects showed a light level of stress in 2020 while 60% in 2021. High levels of stress were shown in 2020 and 2021 for 68% and 72% of FR and 56% and 60% of SR subjects, respectively. Differences between the groups were assessed as statistically significant (*H* = 13.451; *r* = 0.622; *p* ≤ 001) (Table 4).

The statistical inference made it clear that mental health problems that may indicate trauma are mainly present in the FR group. These symptoms decreased slightly in comparison between periods 2020 and 2021 (*p* < 0.05).

## 4. Discussion

Mental health topics continue to be downplayed socially. The COVID-19 pandemic, as noted in the introduction of this paper, has only highlighted the concerns about the human mental sphere that have been noted for years. To date, many studies have been conducted confirming the variables discussed in the research section (depressive symptoms, anxiety, and stress). However, there are few studies that comprehensively approach these three aspects, like the nonetheless study. Although the authors observed a decrease in the indicators studied, these are not drastic changes for the better. The quality of life and mental health of people exposed to a decline in mental form due to being in a stressful social context (COVID-19 pandemic) should be monitored. Which is confirmed by the studies of other scientists cited below.

COVID-19 has had a significant impact on mental health particularly of frontline HCWs. In the study by Yang et al. [37], the majority of emergency nurses had experience attending to COVID-19 patients who were either suspected or confirmed. During the two waves of the survey, which were conducted in the sixth and ninth months of COVID-19, they reported the same levels of stress. According to them, stress levels were higher than they were before the COVID-19 outbreak, and the first three months of the pandemic had the highest levels of stress compared to the sixth and ninth months. While there were significant declines in the reporting of depressive symptoms, this study found that during the three-month follow-up, more than 50% of nurses still reported symptoms of irritability, emotional lability, and body tension. A review study by Cabarkapa et al. [16] noted that nurses who worked in frontline positions and came into touch with suspected and confirmed COVID-19 patients were at a greater risk of developing mental health issues. About 20% of HCWs reported PTSD symptoms 1 month after the MERS epidemic [26] and 2 months after the SARS outbreak [27] according to earlier research with a retrospective methodology. According to a recent review study, emergency unit HCWs had significant PTSD rates during the COVID-19 peak period [28]. Yang et al.'s [37] study reveal that with 3-month prospective observations, a large percentage (at least 30%) of emergency nurses continued to be at risk of developing PTSD cases after being exposed to stress from the COVID-19 epidemic for 9 months, similar to the previous study on HCWs [29]. To stop people from developing PTSD, the psychological treatment must therefore specifically target this high-risk group. In line with the review studies [16, 37], it is important to point out that common stressors like a lack of PPE remained constant over the course of the two-wave period of the COVID-19 pandemic. The Yang et al. study [37] also discovered that the main causes of anxiety level events for emergency nurses were patient and family issues such as hiding their breaking infection control regulations and mental discomfort.

In a meta-analysis evaluating the psychological and mental impact of COVID-19 on medical staff and the general population, the prevalence of anxiety was found to be rather similar between these groups (26% and 32%, respectively) [38]. In China, the general public reported experiencing anxiety symptoms in 28.8% of cases and depression symptoms in 16.5% of cases during the early stages of the COVID-19 pandemic [5]. The incidence of anxiety and depression, however, was observed to be about 44% and 50%, respectively, among HCPs [39, 40]. In the Pataka et al. study [41], it was discovered that HCPs' mental health was much worse during the second wave than it was during the first, with anxiety symptoms being reported by 32.8% of participants and depression symptoms by 37.7%. With 23.8% of primary care HCPs reporting symptoms of anxiety and 27.5% of depression, this was more obvious in comparison to the other groups of HCPs. Anxiety symptoms were also found to be more frequent than depression in other studies [40]. According to the available research, the COVID-19 pandemic had a higher than average prevalence of psychological symptoms in HCPs compared to earlier epidemics [42–45]. The Pataka et al. study [41] found that during the second wave, participants felt more angry and lonely compared to the first, with 22% presenting clinically significant anger and 54.3% indicating significant loneliness. This may harm the provision of health services [46]. An evaluation of COVID-19's effects on the mental health of HCPs in 31 countries revealed that those with less social support experienced larger psychological effects, maybe as a result of a lack of opportunities for emotional expression [45]. Also in the Pataka et al. study [41], loneliness was found to be an important factor affecting sleep quality, especially during the second wave in the general population and separately in the baseline HCP group.

The prevalence of sleep problems in COVID-19 medical staff was higher than in other population groups, probably as a result of stress, which is a well-known source of sleep disruptions among HCPs [47]. HCPs in Wuhan reported significant levels of sadness, anxiety, rage, fear, and stress as a result of the risk of infection, close contact with sick people, and the intense workload [48]. In addition, it was discovered that during the pandemic, 39.2% of HCPs in China experienced sleep problems [40]. This crucial reciprocal relationship was further confirmed by the Patak et al. study [41], which discovered a substantial correlation between sleep quality (SCI) and mental health (PHQ4), even in a separate investigation of depression and anxiety.

Huanga and Zhao [48] found that the combined prevalence among first responders for medical emergencies during the COVID-19 pandemic was 31% for depression, 32% for anxiety, and 17% for stress. Mild forms of depression, anxiety, and stress are more common than moderate and severe forms. According to the results of the current study, sadness, anxiety, and stress are more common than they were before the COVID-19 pandemic [25]. In a recent meta-analysis study, Petrie et al. [49] discovered that 15% of ambulance workers had depression and/or anxiety combined. Additionally, a recent meta-analysis study by Fan et al. [50] found that 19.4% of healthcare workers had depression during the SARS and MERS epidemics. It is probable that the COVID-19 pandemic's greater worldwide impact than the MERS and SARS epidemics, including the number of infected patients and deaths, is the cause of the higher rates of depression, anxiety, and stress among first responders during the COVID-19 pandemic. Additionally, because they work in uncontrolled situations (such as patients' homes, offices, and public spaces), first responders may have seen a greater number of patients who were infected with or who had passed away from COVID-19. The COVID-19 pandemic's scarce protective resources and increased workload may have also had an impact on them. Therefore, providing work resources (such as protective gear) to ensure and enhance first responders' safety may eventually result in higher psychological well-being and prehospital medical service delivery [51].

It is also worth mentioning that the study by Wild et al. [52] emphasizes the need of mentioning index trauma events and the emergence of symptoms when discussing psychopathology connected to the pandemic. It is possible that preexisting PTSD contributed to the elevated incidence of PTSD observed in this and other studies of frontline healthcare workers conducted during the epidemic. The study in question discovered that the onset of major depression was most likely to take place during the pandemic, which is contrary to the pattern seen with PTSD but consistent with the pattern of increased depression in the general population during the pandemic [53–55].

## 5. Strengths and Limitations

Undoubtedly, the strength of the above study is its pioneering nature, which included several variables (signs of reduced quality of life and mental health as a result of the COVID-19 pandemic) and a large study group. In addition, the study was conducted on the basis of scientifically recognized questionnaires, scales, and inventories, which are standardized tools with proven psychometric validity. In addition, the authors made every effort to minimize, occurring in questionnaire studies, systematic error—safeguards were applied for fake/bot responders, times of logging in and completing questionnaires were checked, and check keys were used. Also, the survey conducted has its limitations, of course. First of all, its indirect nature may have influenced the respondents' answers, but it is worth noting that the authors, as described above, made every effort to minimize possible bias. In addition, when using psychometric scales, it is always worth bearing in mind that they are only part of a diagnostic investigation, not a diagnosis in itself, and can only speak to the possibility of a phenomenon and a certain increased risk and not give a full picture of the situation. In addition, what should be noted, the study did not take into account additional variables that will be worth evaluating in further studies.

## 6. Conclusions

According to the current study, the COVID-19 pandemic significantly increased the prevalence of depression, anxiety, and stress among first responders. To ensure the psychological well-being of first responders, early assessment and care of mild depression, anxiety, and stress should be promoted to prevent the development of moderate and severe forms. To avoid both the immediate and long-term effects of these detrimental psychological outcomes, first responders should have access to essential support programs and interventions for the management of depression, anxiety, and stress. Future high-quality research with bigger sample sizes and information on personal and professional characteristics should be encouraged to have a fuller picture of the nature of depression, anxiety, and stress in first responders.

## Figures and Tables

**Table 1:** BDI results (*N* = 300).

BDI	FR		SR	
	2020	2021	2020	2021
Lack of depression	40%	32%	50%	45%
Mild depression	58%	66%	44%	51%
Moderate depression	6%	4%	2%	2%
Severe depression	0%	0%	0%	0%
*p* value	**≤001**		**≤001**	

^∗^ *p* values for the result of the ANOVA Kruskal-Wallis statistical test—comparison between 2020 and 2021 periods.

**Table 2:** GAD-7 results (*N* = 300).

GAD-7	FR		SR	
	2020	2021	2020	2021
Lack of anxiety	18%	28%	45%	45%
Mild anxiety	12%	12%	30%	34%
Moderate anxiety	14%	12%	10%	6%
Severe anxiety	16%	10%	4%	6%
Generalized anxiety (greater than 10 points)	40%	38%	11%	9%
*p* value	**≤001**		**≤001**	

^∗^ *p* values for the result of the ANOVA Kruskal-Wallis statistical test—comparison between 2020 and 2021 periods.

**Table 3:** FCV-19S results (*N* = 300).

FCV-19S	Group	*X*	SD	MIN	MAX	Me	Mo	*p* value^∗^
I am very afraid of SARS-CoV-2 (coronavirus).	FR	4.1	0.9	1	5	3	4	**≤001**
	SR	2.8	0.7	1	5	3	3	

I feel anxious when I think about coronavirus.	FR	3.5	0.5	1	5	3	4	**≤001**
	SR	2.2	0.7	1	5	3	2	

My hands sweat when I think about coronavirus.	FR	1.9	0.9	1	5	3	2	**≤001**
	SR	1,2	0,9	1	5	3	2	

I'm afraid of losing my life due to coronavirus.	FR	2.5	0.7	1	5	3	2	**≤001**
	SR	1,2	0.5	1	5	3	2	

When I watch the news and learn about coronavirus-related stories on social media, I get nervous or anxious.	FR	3.3	0.5	1	5	3	3	**≤001**
	SR	1.5	0.5	1	5	3	2	

I cannot sleep because I'm worried about getting infected with coronavirus.	FR	2.5	0.3	1	5	3	3	**≤001**
	SR	1.1	0.5	1	5	3	1	

My heart beats rapidly when I think of coronavirus infection.	FR	2.3	0.9	1	5	3	2	**≤001**
	SR	1.1	0.5	1	5	3	1	

Raw score (points)	FR	20.1 ± 4.7 (57%)						**≤001**
	SR	10.6 ± 4.3 (30%)						
Interpretation	FR	Moderate level of anxiety						
	SR	Low anxiety						

^∗^ *p* values for the result of the ANOVA Kruskal-Wallis statistical test—comparison between the FR and SR groups.

**Table 4:** PSS-10 results (*N* = 300).

PSS-10	FR		SR	
	2020	2021	2020	2021
Low perceived stress	32%	28%	44%	40%
High perceived stress	68%	72%	56%	60%
*p* value	**≤001**		**≤001**	

^∗^ *p* values for the result of the ANOVA Kruskal-Wallis statistical test—comparison between 2020 and 2021 periods.

## Data Availability

Data is available on request from authors.
